# Glutamine Therapy Reduces Inflammation and Extracellular Trap Release in Experimental Acute Respiratory Distress Syndrome of Pulmonary Origin

**DOI:** 10.3390/nu11040831

**Published:** 2019-04-12

**Authors:** Gisele Pena de Oliveira, Jamil Zola Kitoko, Phillipe de Souza Lima-Gomes, Natália Cadaxo Rochael, Carla Cristina de Araújo, Pâmella Nowaski Lugon, Heloísa Lopes dos Santos, Eduarda Gabrielle Lopes Martins, Felipe Mateus Ornellas, Helena D’Anunciação de Oliveira, Marcelo Marcos Morales, Priscilla Christina Olsen, Antônio Galina, Pedro Leme Silva, Elvira Maria Saraiva, Paolo Pelosi, Patricia Rieken Macedo Rocco

**Affiliations:** 1 Laboratory of Pulmonary Investigation, Carlos Chagas Filho Institute of Biophysics, Federal University of Rio de Janeiro, Rio de Janeiro 21941-902, Brazil; giselepoliv@gmail.com (G.P.d.O.); jamilkitoko@hotmail.com (J.Z.K.); dearaujo.carlacristina@gmail.com (C.C.d.A.); pamellalugon@yahoo.com.br (P.N.L.); heloisalopes_s2@hotmail.com (H.L.d.S.); pedro.leme@gmail.com (P.L.S.); 2 Laboratory of Clinical Bacteriology and Immunology, Department of Toxicological and Clinical Analysis, Faculty of Pharmacy, Federal University of Rio de Janeiro, Rio de Janeiro 21941-902, Brazil; priscillachristinaolsen@gmail.com; 3 Laboratory of Leishmaniasis Immunobiology, Immunology Department, Paulo de Góes Microbiology Institute, Federal University of Rio de Janeiro, Rio de Janeiro 21941-902, Brazil; lima.phillipe@gmail.com (P.d.S.L.-G.); natyrochael@yahoo.com.br (N.C.R.); esaraiva@micro.ufrj.br (E.M.S.); 4 Laboratory of Bioenergetics and Mitochondrial Physiology, Institute of Medical Biochemistry Leopoldo de Meis, Federal University of Rio de Janeiro, Rio de Janeiro 21941-902, Brazil; eduarda_gabrielle@hotmail.com (E.G.L.M.); galina@bioqmed.ufrj.br (A.G.); 5 Laboratory of Cellular and Molecular Physiology, Carlos Chagas Filho Institute of Biophysics, Federal University of Rio de Janeiro, Rio de Janeiro 21941-902, Brazil; fmornellas@gmail.com (F.M.O.); hdanunciacao@gmail.com (H.D.d.O.); mmorales@biof.ufrj.br (M.M.M.); 6 Department of Surgical Sciences and Integrated Diagnostics (DISC), University of Genoa, 16132 Genoa, Italy; ppelosi@hotmail.com; 7 IRCCS Policlinico San Martino Hospital, 16132 Genoa, Italy

**Keywords:** glutamine, pulmonary acute respiratory distress syndrome, lung mechanics, extracellular traps, reactive oxygen species

## Abstract

The innate immune response plays an important role in the pathophysiology of acute respiratory distress syndrome (ARDS). Glutamine (Gln) decreases lung inflammation in experimental ARDS, but its impact on the formation of extracellular traps (ETs) in the lung is unknown. In a mouse model of endotoxin-induced pulmonary ARDS, the effects of Gln treatment on leukocyte counts and ET content in bronchoalveolar lavage fluid (BALF), inflammatory profile in lung tissue, and lung morphofunction were evaluated in vivo. Furthermore, ET formation, reactive oxygen species (ROS) production, glutathione peroxidase (GPx), and glutathione reductase (GR) activities were tested in vitro. Our in vivo results demonstrated that Gln treatment reduced ET release (as indicated by cell-free-DNA content and myeloperoxidase activity), decreased lung inflammation (reductions in interferon-γ and increases in interleukin-10 levels), and improved lung morpho-function (decreased static lung elastance and alveolar collapse) in comparison with ARDS animals treated with saline. Moreover, Gln reduced ET and ROS formation in BALF cells stimulated with lipopolysaccharide in vitro, but it did not alter GPx or GR activity. In this model of endotoxin-induced pulmonary ARDS, treatment with Gln reduced pulmonary functional and morphological impairment, inflammation, and ET release in the lung.

## 1. Introduction

The acute respiratory distress syndrome (ARDS) is characterized by diffuse lung inflammation, which results in endothelial and epithelial damage and a subsequent increase in vascular permeability [1,2]. The innate immune response plays an important role in the pathophysiology of ARDS [3]. Neutrophil influx into the lungs, which is associated with the severity of ARDS, promotes tissue injury and sustains inflammation [4]. Inappropriate accumulation and activation of neutrophils in the alveoli, together with delayed apoptosis, promote unrestrained release of reactive oxygen species, proteases, and neutrophil extracellular traps (NETs) [5,6]. NETs are web-like structures consisting of a decondensed neutrophil chromatin scaffold anchoring antimicrobial proteins, such as neutrophil elastase (NE), myeloperoxidase (MPO), and α-defensins, extruded to the extracellular milieu, which can trap and kill pathogens [7,8]. However, it may also damage lung tissue and, thus, play a role in ARDS pathogenesis [9]. Since no pharmacological approaches are effective in ARDS, modulation of NET formation is an appealing target for adjuvant therapy. 

In this context, several experimental studies have investigated the effects of glutamine (Gln) in pulmonary ARDS [10,11,12,13]. Some showed that Gln can reduce neutrophil infiltration in the lungs [10,11]. Others detected increased inflammatory cytokine production and neutrophil recruitment in early-stage ARDS [12], while some found no effect on neutrophil recruitment into the lung [13]. Whether Gln could help reduce the buildup of NETs in pulmonary ARDS is unknown. 

In the present study, we tested the effects of Gln on the release of extracellular traps (ETs) from cells in the alveolar space in a mouse model of endotoxin-induced pulmonary ARDS. We chose to use the term “ET” instead of “NET” throughout since different cellular types in addition to neutrophils may release extracellular traps [14]. We were unable to identify the origin of these extracellular traps in vivo. We also evaluated the formation of ETs in vitro and the mechanisms that could be involved in the Gln modulation of ETs formation. in vivo, we observed that administration of Gln to animals with endotoxin-induced pulmonary ARDS was associated with a reduction in ET formation and appeared to mitigate lung inflammation and morpho-functional impairment. Moreover, Gln decreased ET and ROS release in BALF cells stimulated with LPS in vitro. 

## 2. Materials and Methods

### 2.1. Ethics Statement

The Animal Welfare Committee of the Health Sciences Center, Federal University of Rio de Janeiro (CEUA-CCS-098/15) approved this study. All animals received humane care in compliance with the “Principles of Laboratory Animal Care” formulated by the National Society for Medical Research and the U.S. National Research Council *Guide for the Care and Use of Laboratory Animals*. 

### 2.2. Animal Preparation and Experimental Protocol

Sixty-one BALB/c mice (weight 20–25 g, age 6 weeks, male and female) were used. The mice were kept under specific pathogen-free conditions, on a 12:12 h light-dark cycle and controlled temperature (20–22 °C), with unrestricted access to food and water. For the in vivo experiments, endotoxin was instilled intratracheally (i.t.) to induce pulmonary ARDS. The mice were randomly divided into two main groups. In the ARDS group, mice received *Escherichia coli* O55:B5 lipopolysaccharide (LPS-B5 Ultrapure, catalog #tlrl-pb5lps, InvivoGen, San Diego, CA, USA) (10 µg in 0.05 mL saline, i.t.), while, in the control group, the animals did not undergo any surgical procedures, instillations, or injections. For intratracheal instillation, mice were anesthetized with sevoflurane. A 1-cm-long midline cervical incision was made to expose the trachea and LPS was instilled using a bent 27G tuberculin needle. The cervical incision was closed with 5-0 silk suture and the mice returned to their cage. All mice received tramadol (0.05 mg/kg body weight by subcutaneous injection) for postoperative analgesia [15]. The animals recovered rapidly after surgery. 

Six hours after LPS instillation, ARDS mice were further randomized to receive an intravenous dose of saline (170 µL, Sal), or Gln (0.75 g/kg body weight, 170 µL, Gln), since L-alanyl-L-glutamine dipeptide (Dipeptiven 20%; Fresenius Kabi Brazil Ltd.a., Campinas, São Paulo, Brazil) resulted in ARDS-Sal and ARDS-Gln, respectively. Saline or Gln were injected into the right jugular vein after induction of inhaled anesthesia with sevoflurane. Similarly, animals in the control group were treated simultaneously with originating subgroups Control-Sal and Control-Gln. To observe the inflammatory process in the lung, at the time of treatment, eight non-treated animals from the control and ARDS groups (*n* = 4/each) were sacrificed with a lethal dose of thiopental sodium (300 µL, intraperitoneally) and the number of leukocytes in bronchoalveolar lavage fluid (BALF) was counted. Experiments were performed in triplicate. The data shown are from a single representative experiment.

Twenty-four hours after induction of ARDS, 24 animals (*n* = 6/group) were selected for evaluation of leukocyte counts in the BALF, visualization of ETs in pulmonary sections, quantitation of inflammatory cytokines in lung tissue (IL-10, IL-1β, and IFN-γ), and assessment of lung function and morphometry. Another subset of animals subjected to the same injury and treatment protocol (*n* = 6/group) was selected for investigation of total leukocyte, neutrophil, and mononuclear cell counts in BALF and quantitation of ETs (cell free-DNA, MPO, and NE) in BALF. 

For the in vitro experiments, an additional group of five healthy female BALB/c mice was used for isolation of leukocytes from the alveolar space. To recruit neutrophils to the alveolar space, 9% casein was instilled i.t. in healthy mice, after induction of inhaled anesthesia with sevoflurane. An incision similar to that used for LPS administration was made. BALF was collected 16 h after casein instillation [16]. Cells isolated from BALF were then stimulated for 3 h with LPS (20 µg/mL), at 35 °C, in Gln-free medium (RPMI Medium 1640—No Glutamine, 12633-012, Thermo Fisher Scientific Inc., Waltham, MA, USA), in the presence or not of added Gln (as L-alanyl-L-glutamine dipeptide, Dipeptiven 20%, Fresenius Kabi Brazil Ltd.a., Campinas, São Paulo, Brazil) at 500 µM. Non-stimulated cells were used as a control. Formation of ETs (cf-DNA), ROS production, and glutathione peroxidase (GPx) and glutathione reductase (GR) enzyme activity were measured.

### 2.3. Total and Differential Cell Counts in BALF

Bronchoalveolar lavage was carried out with phosphate-buffered saline solution (3 × 400 µL). BALF was centrifuged at 4 °C for 10 min at 400× *g* and the cell pellet resuspended in phosphate-buffered saline (250 µL) for further leukocyte enumeration. The supernatant was stored (−80 °C) for subsequent analysis of cf-DNA, MPO, and elastase. Total leukocyte counts were measured in a Neubauer chamber under light microscopy after diluting the samples in Türk’s solution (Merck KGaA, Darmstadt, Germany). For differential cell counts (mononuclear cells and neutrophils), cytospin slides were prepared and stained by the panoptic method (Laborclin, Pinhais, PR, Brazil) [17]. 

### 2.4. Identification of ETs in BALF: Quantification of cf-DNA, Elastase, and Myeloperoxidase Activity

The supernatants from each sample obtained after BALF centrifugation were distributed into 96-well opaque plates and cf-DNA was quantified using the Quant-iT PicoGreen dsDNA Assay Kit (Thermo Fisher Scientific Inc., Waltham, MA, USA), read in a spectrofluorometer (Spectra-Max^®^ Paradigm^®^ microplate reader, Molecular Devices, Sunnyvale, CA, USA) at 485/538 nm excitation/emission [18]. 

To quantify elastase activity, supernatants (25 µL) were incubated with the elastase substrate *N*-methoxysuccinyl-Ala-Ala-Pro-Val-7-amido-4-methylcoumarin (0.25 mM, Sigma) in buffer (50 mM HEPES, 100 mM NaCl, 0.01% Triton X-100) for 30 min at 37 °C and 5% CO_2_, protected from light. The reaction product was analyzed at 360/455 nm. MPO activity was assayed in BALF supernatants (7 µL) incubated with hexadecyltrimethylammonium bromide (HTAB, 60 µL), tetramethylbenzidine (TMB, 4 µL), and hydrogen peroxide (H_2_O_2_, 40 µL). After 60 min at 37 °C, the colorimetric reaction was interrupted by adding sodium acetate (2M). The reaction product was analyzed at 630 nm on a spectrophotometer [18]. 

### 2.5. Enzyme-Linked Immunosorbent Assay (ELISA)

IL-1β, IL-10, and IFN-γ (PeproTech, Rocky Hill, NJ, USA) levels were quantified in left-lung homogenates using commercially available ELISA kits, in accordance with manufacturer instructions. Lung tissue was homogenized in lysis buffer (250 mM sucrose, 20 mM HEPES, 1 mM EDTA, 50 mM NaF, 1 mM phenylmethylsulfonyl fluoride, 1× Roche protease inhibitor cocktail [Roche Diagnostic, Mannheim, Germany]) using a glass Potter tissue grinder with a Teflon^®^ piston. The total cytokine content was normalized to total protein content, which was quantified by using Bradford’s reagent (Sigma-Aldrich, St Louis, MO, USA).

### 2.6. Lung Mechanics 

To investigate the effects of Gln on lung function during pulmonary ARDS, we analyzed lung mechanics 24 h after LPS instillation. ARDS and control mice treated with saline or Gln were sedated (diazepam 1 mg/kg, *i.p.*), anesthetized (ketamine 67 mg/kg and xylazine 30 mg/kg, *i.p.*), tracheotomized, paralyzed (vecuronium bromide, 0.005 mg/kg, *i.v.*), and ventilated with a constant-flow ventilator (Samay VR15; Universidad de la Republica, Montevideo, Uruguay). The ventilator was set to a respiratory frequency of 100 breaths.min^−1^, tidal volume (V_T_) 0.2 mL, and fraction of inspired oxygen (FiO_2_) 0.21. The anterior chest wall was surgically removed and a positive end-expiratory pressure (PEEP) of 2 cmH_2_O was applied to avoid alveolar collapse.

After a 10-min ventilation period, airflow and tracheal pressure (Ptr) were measured. In an open chest preparation, Ptr reflects transpulmonary pressure (P_L_). Static lung elastance (Est,L) was determined by dividing the elastic recoil pressure of the lung (Pel) by V_T_, using the end-inflation occlusion method [19].

Data were analyzed using ANADAT data analysis software (RHT-InfoData, Inc., Montreal, QC, Canada). All experiments lasted less than 20 min.

### 2.7. Lung Histology

To support mechanical data, the structure of the lung parenchyma was evaluated by light microscopy. Immediately after determination of lung mechanics, a laparotomy was performed and heparin (1000 IU) was injected into the vena cava. The trachea was clamped at end-expiration (PEEP = 2 cmH_2_O) and the abdominal aorta and vena cava were sectioned, which yielded a massive hemorrhage that quickly killed the animals. The right lung was then removed, fixed in 4% buffered formalin, and paraffin-embedded. Sections were cut (4 µm thick) and stained with hematoxylin and eosin (Vetec Química Fina, Rio de Janeiro, Brazil). 

The volume fractions of collapsed and normal pulmonary areas were determined by the point-counting technique at a magnification of ×200 across 10 random, noncoincident microscopic fields [17].

### 2.8. Detection of ETs (cf-DNA) in Vitro

Bronchoalveolar lavage was carried out in mice that underwent intra-tracheal instillation of 9% casein, as previously described. Total leukocyte numbers were measured in a Neubauer chamber under light microscopy after diluting the samples in Türk’s solution. Differential cell counts (mononuclear and neutrophils) were performed in cytospin slides stained by the panoptic method (Laborclin, Pinhais, PR, Brazil).

As described above, BALF cells (2.5 × 10^5^) were re-suspended in Gln-free RPMI medium and incubated with 20 µg/mL LPS in the presence or absence of 500 µM Gln. ETs were quantified as cf-DNA in the culture supernatant using the Picogreen dsDNA kit, according to the manufacturer’s instructions. In addition, ETs were also detected in BALF cells (2.5 × 10^5^) plated in poly L-lysine treated coverslips, using the same protocol described above. The cells were fixed in 4% buffered formalin overnight at 4 °C, washed thoroughly, and incubated with anti-citrullinated histone H3 antibody (Cit-H3, 1:250, ab5103, Abcam), which was followed by anti-rabbit IgG F(ab′)2 Cy3 antibody (1:100, C2306, Sigma Aldrich, St Louis, MO, USA). In addition, 4′,6-diamidino-2-phenylindole (DAPI) was applied to detect DNA. ETs were identified based on the co-localization of DNA with Cit-H3. ETs images in fifteen high-power field (×200) per slide were visualized in an Apotome.2 microscope (Zeiss, Germany) and were then analyzed in Image-Pro Plus 6.3 for Windows software (Media Cybernetics, Silver Spring, MD, USA). Fluorescence microscopy images were analyzed to count the number of positive cells to Cit-H3/DNA co-localization per 100 cells.

### 2.9. Reactive Oxygen Species Measurement

As described above, BALF cells (2.5 × 10^5^) were distributed into 96-well opaque plates (2.5 × 10^5^/well) in Gln-free RPMI medium in the presence or absence of 500 µM Gln. The cells were then stimulated with LPS (20 μg/mL) or phorbol myristate acetate (PMA, 100 nM, Merck, Darmstadt, Germany) as a positive control. Then, dihydrorhodamine (DHR) 123 (1.2 μM; Sigma-Aldrich, St Louis, MO, USA) was added, and ROS production was measured every 10 min for 3 h at 35 °C, using 530/590 nm excitation/emission, on a Spectra-Max^®^ Paradigm^®^ microplate reader (Molecular Devices, San Jose, CA, USA).

### 2.10. Determination of Glutathione Reductase and Glutathione Peroxidase Activity

GPx activity was measured indirectly by monitoring the oxidation of NADPH [20]. A reaction mixture containing 0.5 mM GSH, cell fraction (5 µg/mL), and 0.24 units/mL GR was preincubated for 10 min at 37 °C in phosphate buffer (0.1 M, pH 7.0) to a final volume of 100 µL. Thereafter, 0.15 mM β-NADPH was added and the hydroperoxide-independent consumption of β-NADPH was monitored for approximately 3 min. The overall reaction was started by adding 1.2 mM of *tert*-butyl hydroperoxide (prewarmed solution). The decrease in absorption at 340 nm was monitored for approximately 5 min. The non-enzymatic reaction rate was correspondingly assessed by replacing the mitochondrial sample with buffer. One unit of GPx is equivalent to the oxidation of 1 µmol of NADPH per min at pH 7.0 and 37 °C.

Monitoring the oxidation of β-NADPH measured GR activity [20]. The reaction mixture contained 1 mM GSSG and 0.1 mM β-NADPH in phosphate buffer (0.1 M, pH 7.0) to a final volume of 1 mL at 30 °C. The reaction was initiated by adding the mitochondrial fraction (50 µg/mL) to the cuvette, and the decrease in absorbance at 340 nm was followed at 30 °C. One unit of GR is equivalent to the oxidation of 1 µmol of NADPH per min at pH 7.0 and 30 °C.

### 2.11. Statistical Analysis 

The sample size was calculated on the basis of pilot studies, which detected differences in Est,L between ARDS-Sal and ARDS-Gln. A sample size of 6 animals per group would provide the appropriate power (1 − β = 0.8) to identify significant differences in Est,L (adjusted α = 0.025 for two comparisons), taking into account an effect size *d* = 2.0, a two-sided *t*-test, and a sample size ratio of 1 (G*Power 3.1.9.2, University of Düsseldorf, Düsseldorf, Germany). 

The normality of the data (Kolmogorov–Smirnov test with Lilliefors’ correction) and the homogeneity of variances (Levene median test) were tested. Parametric data are expressed as mean ± SD, while nonparametric data are expressed as median (interquartile range). Differences among the groups were assessed by two-way ANOVA followed by Bonferroni’s test. Nonparametric data were analyzed using ANOVA on ranks followed by Dunn’s test. All tests were performed in the GraphPad Prism v6.00 software environment (GraphPad Software, La Jolla, CA, USA). Significance was established at *p* < 0.05.

## 3. Results

### 3.1. In Vivo Experiments

#### 3.1.1. Leukocyte Recruitment in BALF

**Figure 1:**
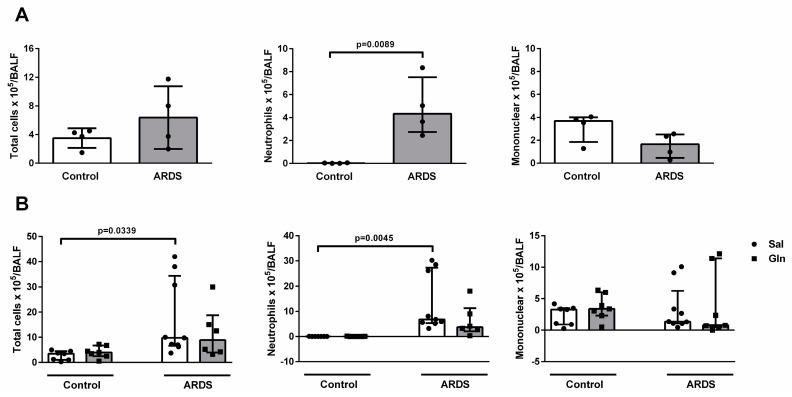
Effects of glutamine on leukocyte recruitment in bronchoalveolar lavage fluid (BALF) in a mouse model of pulmonary ARDS. (**A**) BALB/c mice were randomly allocated to control or acute respiratory distress syndrome (ARDS) groups. In ARDS, mice received *E. coli* LPS intratracheally (10 µg/0.05 mL saline), while control animals did not undergo any surgical procedures, instillations, or injections. Six hours after LPS administration, animals in the control and ARDS groups (*n* = 4/each) were sacrificed for leukocyte enumeration and differential cell counts in BALF. (**B**) In another group of control and ARDS animals, mice were treated 6 h after LPS instillation with an intravenous dose of saline (170 µL, Sal) or Gln (0.75 g/kg body weight, 170 µL). Twenty-four hours after LPS instillation, Control-Sal, Control-Gln, ARDS-Sal, and ARDS-Gln animals were sacrificed for leukocyte enumeration and differential cell counts in BALF. Values are presented as median (interquartile range). Symbols represent individual animals. Experiments were performed in triplicate. The data shown are from a single representative experiment.

To evaluate leukocyte recruitment in the lung at the time of treatment, non-treated animals in the ARDS group were sacrificed and BALF was collected 6 h after LPS instillation. BALF was also collected in control mice at the same time. Six hours after LPS, total as well as mononuclear cell counts were not significantly different between control and ARDS groups (3.5 ± 1.37 × 10^5^ vs. 6.37 ± 4.38 × 10^5^ and 3.18 ± 1.28 × 10^5^ vs. 1.54 ± 1.09 × 10^5^, respectively). The neutrophil count was higher in ARDS (4.86 ± 2.55 × 10^5^) compared to control animals (0.02 ± 0.02 × 10^5^) (Figure 1A). In another group of animals, Gln or saline was administered at 6 h and BALF was collected 24 h after LPS instillation. Total cells and neutrophils were higher in ARDS-Sal than in Control-Sal animals (17.19 ± 15.18 × 10^5^ vs. 2.75 ± 1.88 × 10^5^ and 13.29 ± 11.37 × 10^5^ vs. 0.01 ± 0.02 × 10^5^, respectively), while mononuclear cell counts were similar between ARDS-Sal and Control-Sal (3.4 ± 3.64 × 10^5^ vs. 2.38 ± 1.57 × 10^5^, respectively). In comparison with Sal, Gln did not change the total, neutrophil, or mononuclear cell counts in either the control or ARDS groups (Figure 1B).

#### 3.1.2. Glutamine Treatment Reduced ET Formation

**Figure 2:**
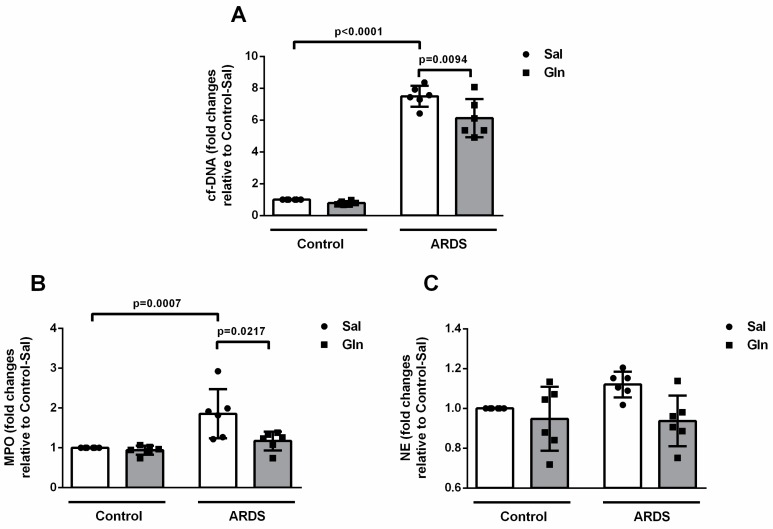
Glutamine decreased ET markers in bronchoalveolar lavage fluid (BALF) in a mouse model of pulmonary ARDS. (**A**) Cf-DNA was quantified using the PicoGreen assay kit in the supernatant from each sample obtained from BALF centrifugation. (**B**) Myeloperoxidase (MPO) activity was assayed in BALF supernatants incubated with hexadecyltrimethylammonium bromide (HTAB), tetramethylbenzidine (TMB), and hydrogen peroxide (H_2_O_2_). (**C**) Neutrophil elastase (NE) activity was measured in BALF supernatants incubated with N-methoxysuccinyl-Ala-Ala-Pro-Val-7-amido-4-methylcoumarin. Values normalized in relation to Control-Sal are expressed as mean ± SD of six animals in each group. Symbols represent individual animals. Experiments were performed in triplicate. The data shown are from a single representative experiment.

Experimental studies have linked ETs to ARDS [21,22,23]. Since the number of mononuclear cells and neutrophils in BALF was not modified by Gln treatment (Figure 1B), we decided to evaluate whether Gln could alter the function of these cells in the alveolar space—specifically, ET formation. LPS instillation increased cf-DNA content 7.5-fold in BALF from ARDS-Sal compared to Control-Sal animals. In ARDS-Gln animals, cf-DNA was 0.81-fold lower than in the ARDS-Sal group (Figure 2A). MPO activity in the BALF supernatant followed changes in cf-DNA. It was 1.86-fold higher in ARDS-Sal compared to Control-Sal animals and 0.63-fold lower in ARDS-Gln than in ARDS-Sal animals (Figure 2B). Neither cf-DNA content nor MPO activity was affected by Gln treatment in the control group. In addition, NE activity in the BALF supernatant was not affected by ARDS induction or Gln treatment (Figure 2C).

#### 3.1.3. Lung Cytokine Modulation by Glutamine Treatment

**Figure 3:**
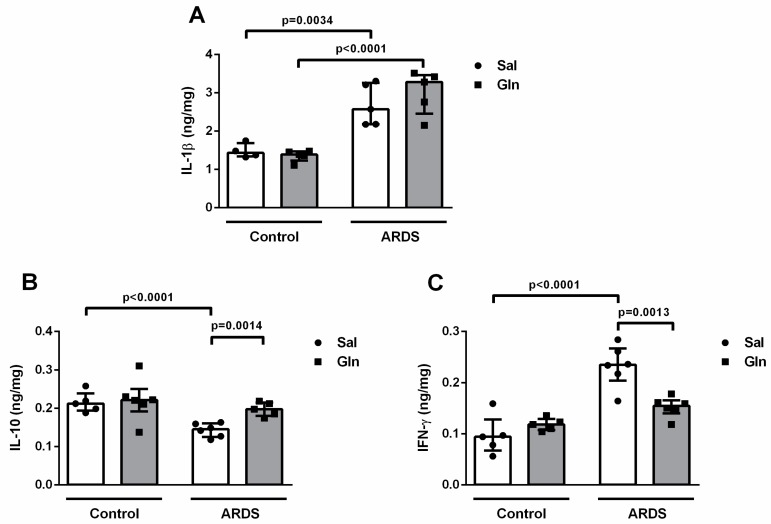
Glutamine in a mouse model of pulmonary ARDS differentially modulated cytokines in lung tissue. The cytokines interleukin (IL)-1β (**A**), IL-10 (**B**), and interferon (IFN)-γ (**C**) were evaluated by specific ELISA in the left lung of Control-Sal, Control-Gln, ARDS-Sal, and ARDS-Gln BALB/c mice (*n* = 6/each). Lung tissue samples were removed after collection of lung mechanics data 24 h after LPS instillation. The total cytokine content was normalized to total protein content. Values expressed as median (interquartile range) of six animals in each group. Symbols represent individual animals. Experiments were performed in triplicate. The data shown are from a single representative experiment.

Analysis of lung tissue revealed that, in our model of intratracheal LPS administration, IL-1β and IFN-γ contents were 1.82 ng/mg and 2.3 ng/mg higher, respectively, in ARDS-Sal animals vs. Control-Sal animals (Figure 3A,C), while IL-10 content was 0.67 ng/mg lower in ARDS-Sal than in Control-Sal (Figure 3B). Additionally, IL-1β content was also higher (1.57 ng/mg) in ARDS-Gln than in Control-Gln animals (Figure 3A). Treatment with Gln reduced IFN-γ levels by 0.65 ng/mg and increased IL-10 levels by 1.43 ng/mg in ARDS-Gln vs. ARDS-Sal animals (Figure 3A,C). However, Gln in the ARDS groups did not modify IL-1β levels (Figure 3A). These data demonstrate that Gln treatment promoted a less inflammatory environment within the lung after ARDS induction.

#### 3.1.4. Effect of Glutamine on Lung Function and Histology in Pulmonary ARDS

**Figure 4:**
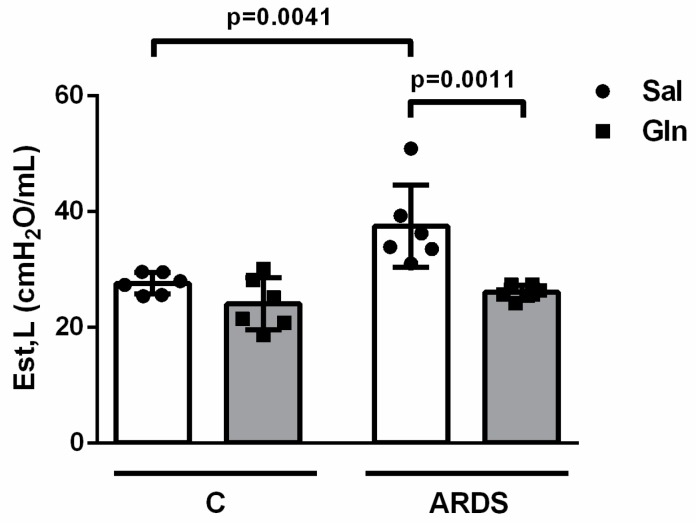
Glutamine reduced lung functional impairment in a mouse model of pulmonary ARDS. Twenty-four hours after ARDS induction, mice were sedated, anesthetized, tracheotomized, paralyzed, and ventilated in constant-flow mode in an open chest preparation. Static lung elastance (Est,L) was measured by the end-inflation occlusion method (10 respiratory cycles/animal). Values in cmH_2_O/mL are expressed as mean ± SD of six animals in each group. Symbols represent individual animals. Experiments were performed in triplicate. The data shown are from a single representative experiment.

Aiming to investigate whether Gln treatment could benefit lung function, we evaluated pulmonary mechanics (Est,L). Administration of LPS caused increased Est,L (37.47 ± 7.13 in ARDS-Sal vs. 27.55 ± 1.85 cmH_2_O/mL in Control-Sal). In the ARDS-Gln group, Est,L was lower than in ARDS-Sal (26.05 ± 1.24 vs. 37.47 ± 7.13 cmH_2_O/mL, respectively) (Figure 4).

**Figure 5:**
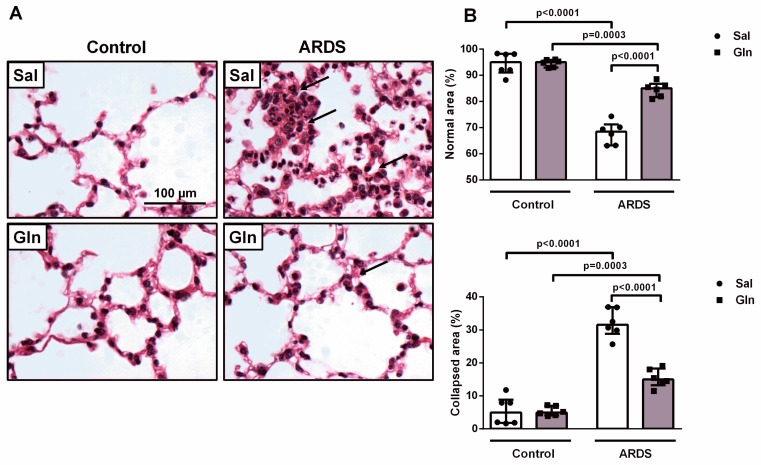
Glutamine administration mitigated histological damage to the lung parenchyma in a mouse model of pulmonary ARDS. At the end of lung mechanics data collection, 24 hours after ARDS induction, animals were sacrificed and the trachea was clamped at the end-expiration (PEEP = 2 cmH_2_O). The right lung was removed, fixed in formalin, and paraffin-embedded. (**A**) Representative photomicrographs of lung parenchyma were stained with hematoxylin and eosin. Bar = 100 µm for all photomicrographs. (**B**) Fractional areas of normal and collapsed alveoli, presented as percentage, were determined by the point-counting technique (10 microscopic fields/slide). Values were expressed as the median (interquartile range) of six animals in each group. Symbols represent individual animals. Experiments were performed in triplicate. The data shown are from a single representative experiment.

Evaluating the lung structure, we observed that LPS instillation induced larger fractional areas of alveolar collapse and, consequently, fewer normal areas in ARDS-Sal (32% and 68%, respectively) compared to Control-Sal animals (6% and 94%, respectively) (Figure 5A,B). A similar behavior was observed in the Gln-treated groups (ARDS-Gln, 15% and 85% of collapsed and normal areas, Control-Gln, 5% and 95% of collapsed and normal areas, respectively) (Figure 5A,B). Gln treatment mitigated parenchymal damage in ARDS-Gln compared with ARDS-Sal (15% and 85% of collapsed and normal areas vs. 32% and 68% of collapsed and normal areas) (Figure 5A,B). Thus, LPS increased heterogeneity in the lung parenchyma, represented by higher alveolar collapse or, in other words, a smaller airspace capable of expanding during inspiration. This heterogeneity may explain the increased Est,L observe d in the ARDS-Sal group.

### 3.2. In Vitro Experiments

#### 3.2.1. Glutamine Treatment Reduced ETs and ROS Formation Induced by LPS in BALF Cells

**Figure 6:**
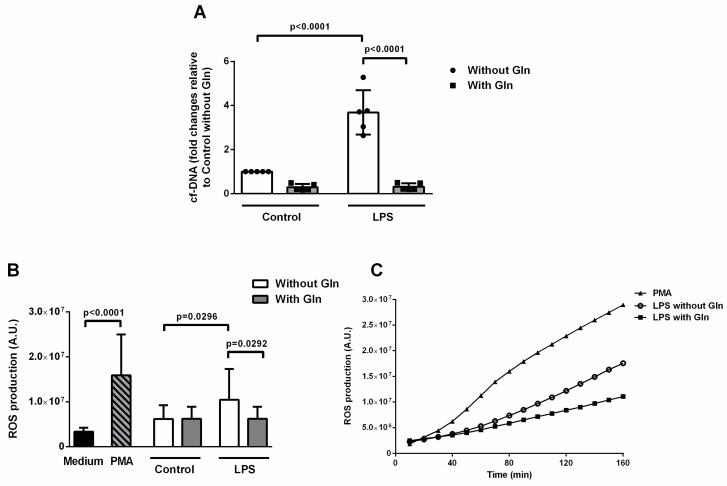
Glutamine decreased cell free (cf)-DNA and ROS production in bronchoalveolar lavage fluid (BALF) cells in vitro. For in vitro experiments, neutrophil recruitment to the alveolar space was induced in six healthy BALB/c animals by intratracheal instillation of 9% casein. BALF cells were distributed into 96-well opaque plates (2.5 × 10^5^/well) in Gln-free medium with or without 500 µM Gln. Cells were stimulated with LPS (20 μg/mL). (**A**) Cf-DNA was quantified after 3 h of LPS stimulation (PicoGreen assay) in the supernatants. Data normalized in relation to control without Gln are expressed as mean ± SD. Symbols represent individual animals. (**B**) ROS production was evaluated on BALF cells collected as above. Dihydrorhodamine (DHR) 123 was used to detect ROS production in LPS-stimulated cells in the presence or absence of Gln for 160 min. PMA 100 nM was used as a positive control. Results expressed as arbitrary units (AU) of ROS production + SD of three experiments. (**C**) Kinetics of ROS production. Experiments were performed in triplicate. The data shown are from a single representative experiment.

In an attempt to better investigate the effects of Gln on ETs release in our model of endotoxin-induced pulmonary ARDS, we performed additional in vitro experiments. Sixteen hours after casein instillation, analysis of BALF leukocytes revealed a total of 2.81 ± 1.21 × 10^5^ and 0.24 ± 0.09 × 10^5^ polymorphonuclear and mononuclear cells, respectively. After stimulating BALF cells with LPS, we found that cf-DNA content was 3.69-fold higher in cells incubated with LPS compared to control cells (Figure 6A). In LPS-stimulated cells, the addition of Gln reduced cf-DNA content 0.09 times in comparison with Gln-free medium (Figure 6A). Similarly, Gln reduced ET release under LPS induced ARDS in vivo (Figure 2A).

Both LPS and PMA induced increased ROS production in the Gln-free medium (69% and 157% higher, respectively) in comparison with the control (unstimulated) cells. On the other hand, cells incubated with LPS in a medium with Gln produced 40% less ROS than cells incubated with LPS but no Gln (Figure 6B). We could observe that, upon LPS or PMA-induced activation, gradually increasing levels of ROS were detectable within minutes after the cell challenge (Figure 6B, *inset*).

**Figure 7:**
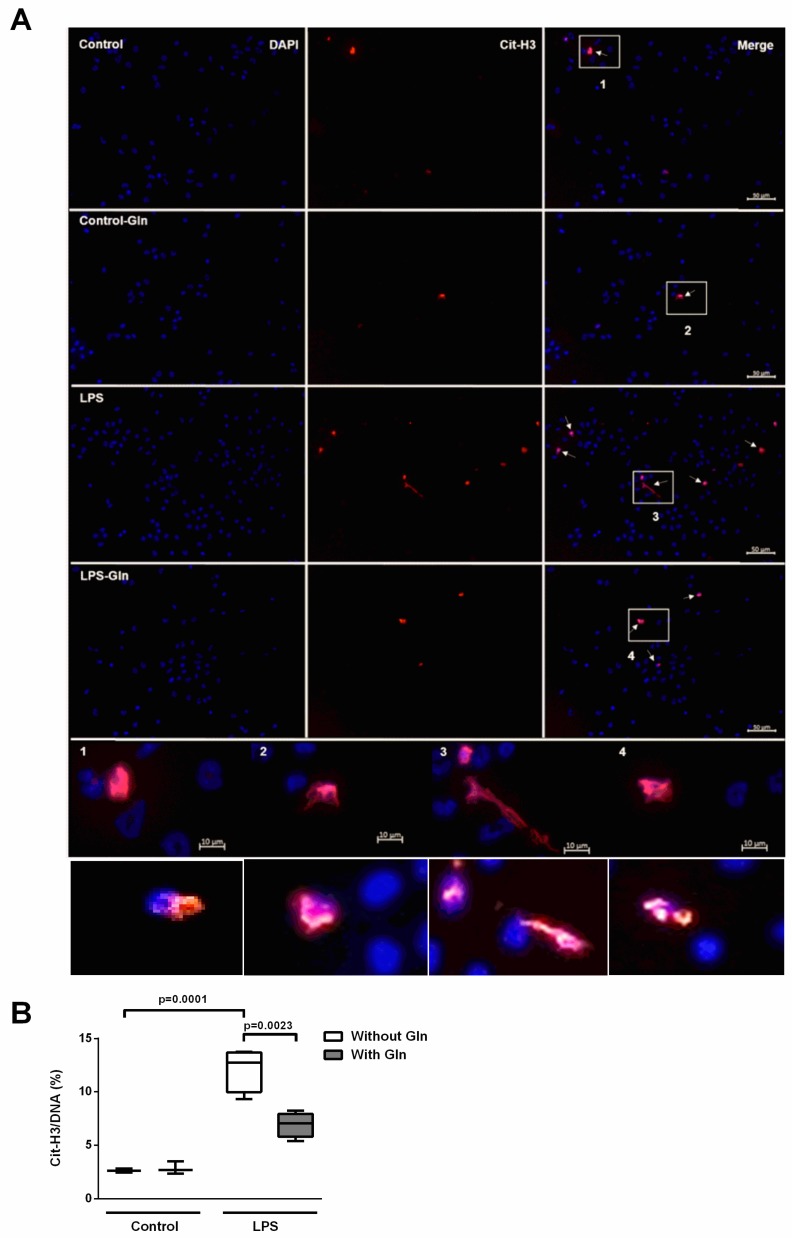
Glutamine reduced Cit-H3/DNA in bronchoalveolar lavage fluid (BALF) cells in vitro. (**A**) Representative immunofluorescence images of plated BALF cells. Staining depicts citrullinated-histone 3 (anti-Cit-H3 antibody, red) and DNA (DAPI, blue). White arrows show ETs. Insets 1, 2, 3, 4: present ET images at higher magnification, demonstrating co-localization of Cit-H3 and DNA. Bottom line shows new images of ETs in each experimental condition. Bars = 50 µm (10 µm for the insets). (**B**) Fluorescence microscopy images were analyzed to count the number of positive cells to Cit-H3/DNA co-localization per 100 cells. Values expressed as median (interquartile range) of six animals in each group. Experiments were performed in triplicate. The data shown are from a single representative experiment.

A surrogate indicator indicated ET formation: co-localization of extracellular DNA with citrullinated-histone H3 (Cit-H3/DNA) by immunofluorescence (Figure 7A). Corroborating our previous findings, LPS-stimulated cells incubated without Gln presented 12% Cit-H3/DNA-positive cells vs. 3% in control cells without Gln. Fewer Cit-H3/DNA-stained cells were observed in cells treated with LPS and Gln than in LPS-stimulated cells without Gln (7% vs. 12%, respectively) (Figure 7A,B). 

#### 3.2.2. Glutathione Reductase and Glutathione Peroxidase Activities

**Figure 8:**
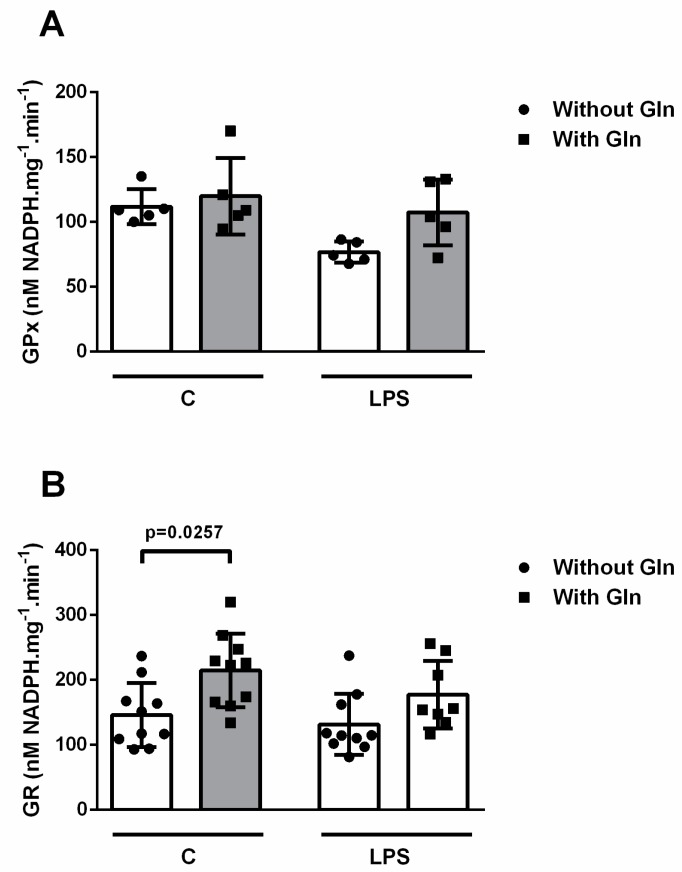
Glutathione reductase (GR) and glutathione peroxidase (GPx) activity in bronchoalveolar lavage fluid (BALF) cells. (**A**) GPx activity was measured indirectly by monitoring NADPH oxidation. One unit of GPx is equivalent to the oxidation of 1 µmol of NADPH per min at pH 7.0 and 37 °C. (**B**) GR activity was measured by monitoring β-NADPH oxidation. One unit of GR is equivalent to the oxidation of 1 µmol of β-NADPH per min at pH 7.0 and 30 °C. Values expressed as mean ± SD of five to 10 animals in each group. Symbols represent individual animals. Experiments were performed in triplicate. The data shown are from a single representative experiment.

As we observed lower ROS production in LPS-stimulated cells in a medium with Gln compared to LPS-stimulated cells in Gln-free conditions, we decided to investigate the redox and antioxidant status of BALF cells. For this purpose, we analyzed the reaction capacity of the enzymes GR and GPx (Figure 8). Neither LPS or Gln (Figure 8A) affected activity of GPx. Control cells incubated in a medium with Gln showed higher GR activity than control cells incubated without Gln (214.8 ± 56.76 vs. 146.2 ± 49.29 nM NADPH/mg.min, respectively) (Figure 8B). However, no difference was observed between control and LPS-stimulated cells incubated without Gln or between LPS-stimulated cells incubated with and without Gln.

## 4. Discussion

Our data, obtained in a model of endotoxin-induced pulmonary ARDS, suggests that modulation of ET release in the lung may be a mechanism of Gln action in this setting. Our in vivo results demonstrated that Gln treatment reduced ET release (as indicated by cf-DNA content and MPO activity), decreased lung inflammation (as indicated by reductions in IFN-γ and increases in IL-10 levels), and improved lung morpho-function (as indicated by decreased Est,L and alveolar collapse) in comparison with saline control (ARDS-Sal). Additionally, both ET and ROS formation were lower when isolated BALF cells were stimulated with LPS in the presence of Gln in vitro. On the basis of these findings, we hypothesized that Gln could reduce ET release through the control of ROS formation. However, Gln did not alter activity of GR or GPx, which are two important enzymes involved in glutathione metabolism and in the intracellular antioxidant defense system. Thus, the mechanisms underlying Gln-mediated reduction of ET formation in pulmonary ARDS still need to be clarified.

The role of Gln in pulmonary ARDS has been studied elsewhere [10,11,12,13]. In rats intratracheally instilled with *E. coli* LPS, early intravenous administration of Gln prevented lung histologic damage, elevated glutathione synthesis, and reduced IL-8 release as well as neutrophil recruitment in the lung [10]. The early use of intravenous Gln also reduced lung neutrophil recruitment and tissue injury in a two-hit model of ventilator-induced lung injury plus acid aspiration in rats [11]. Conversely, higher inflammatory cytokine production and increased neutrophil recruitment in early-stage ARDS were observed in C57BL/6 mice pre-treated for 10 days with a Gln-supplemented diet and subjected to ARDS induction by i.t. instillation of *E. coli* LPS [12]. Another study evaluated the effects of two preventive doses of Gln administered by oral gavage in a rat model of IL-1/LPS instillation and found that Gln activated the anti-inflammatory CD163/heme oxygenase (HO)-1/p38-MAPK dephosphorylation pathway in alveolar macrophages despite unchanged neutrophil recruitment into the lung [13]. Our data showed that treatment with Gln did not modify neutrophil or mononuclear cell counts in BALF. These controversial results could be explained by differences in the route of Gln administration, the model of lung injury, and the timing of treatment, as well as in data collection. Unlike previous studies, we administered Gln once the inflammatory process was already established (as demonstrated by higher neutrophil counts in BALF 6 h after LPS instillation) rather than preventively. Thus, in our setting, Gln did not modify the number of neutrophils in alveolar spaces. We, thus, decided to investigate whether it might alter cell function. 

The release of extracellular traps was first described in neutrophils, which led to the denomination “NET” [7]. During inflammation, neutrophils become activated upon stimulation and may phagocytose, degranulate, extrude NETs, and produce ROS, among other functions. The great majority of stimuli described as inducers of NETs formation are dependent on ROS production by the multi-enzyme complex NADPH oxidase [24]. Therefore, drugs that inhibit NADPH oxidase also inhibit NET release. The structure of NETs is web-like and consists of a de-condensed neutrophil chromatin scaffold anchoring antimicrobial proteins such as neutrophil elastase (NE), myeloperoxidase (MPO), and α-defensins, extruded to the extracellular milieu [7,8]. The evaluation of separated components of extracellular traps has been used in the current literature [18]. In our in vivo analysis, we assessed three different components of extracellular traps: cell-free DNA, NE, and MPO. In our study, ARDS-Sal mice exhibited high cf-DNA content and MPO activity, while elastase activity was not modulated. The ARDS-Gln groups showed lower cf-DNA content and MPO activity in BALF 24 hours after LPS instillation. We did not analyze ETs in our paraffin-embedded lung tissue slices, since the heat-induced epitope retrieval required for successful immuno-histological staining may cause binding to nonspecific proteins. In addition, since we could not define specifically which cell released the cf-DNA and MPO, we chose to use the term ETs rather than NETs to describe our results. Emerging evidence suggests that mature macrophages also release NET-like structures currently known as “METs” [25].

Moreover, we observed lower levels of IFN-γ and higher levels of IL-10 in lung tissue in ARDS mice treated with Gln, but Gln did not affect IL-1β. Studies have demonstrated that IFN-γ, upon subsequent stimulation with complement factor 5a (C5a), acts as a priming factor on mature neutrophils to allow ET formation [26]. Overproduction of IL-10 suppresses the ROS-dependent generation of NETs induced by the human immunodeficiency virus (HIV)-1 [27]. A study demonstrated that, during the course of pneumonias, the great majority of cells expressing IFN-γ were neutrophils, and that IFN-γ regulated bacterial clearance by regulating the production of NETs [28]. Additionally, evidence has shown that the production of oxidants by NADPH oxidase leads to the production of IFN-γ [29]. Thus, targeting neutrophil function, particularly the production of IFN-γ, will modulate specific immune processes such as ETosis. In this line, our result suggests that Gln could be used to modulate IFN-γ production in pulmonary ARDS. Presently, the effects of these mediators on ET formation in ARDS are unknown. In a similar model of LPS-induced pulmonary ARDS in rats, early intravenous Gln treatment prevented morphological changes in the lung parenchyma, enhanced reduced glutathione (GSH) synthesis, attenuated IL-8 release in lung tissue, and prevented higher CD11b expression in blood neutrophils [10]. In a model of experimental ARDS induced by the *i.t.* administration of hydrochloric acid plus *E. coli* LPS, pretreatment with a Gln-supplemented diet reduced levels of the receptor for advanced glycation end-products (RAGE) and IL-1β in BALF [30]. In a two-hit model of hydrochloric acid aspiration plus injurious mechanical ventilation, early intravenous Gln improved lung morpho-function and cytokine production in lung tissue [11]. Therefore, our differences in cytokine findings as compared with previous studies might be related to the timing of treatment and data collection (24 h after ARDS induction), the insult model employed, and the route of Gln administration. 

To evaluate whether Gln treatment reduced ETs by diminishing ROS formation, we performed in vitro experiments in which we recruited neutrophils into the alveolar space by intratracheal casein administration, as previously described [16]. To estimate ET formation, cf-DNA was measured in supernatants using PicoGreen. In the in vitro experiments, we used a 500-µM dose of Gln, which is very similar to its physiological range in plasma (600 µM), since a high Gln dose could result in high intracellular glutamate content and promote toxic effects. High glutamate exposure could interfere with the functional capacity of BALF cells and reduce ROS production, as well as ET release. Previous studies have used very high, non-physiologic doses of Gln (about 2000 µM) for the in vitro cell culture [31]. The LPS stimulus on these casein-recruited BALF neutrophils induced high ET release, while incubation of these cells with LPS in the presence of Gln reduced ET release. Unfortunately, we cannot extrapolate an in vitro result to in vivo conditions, since the elements of the alveolar microenvironment interact to mediate the effect of LPS on the lung [32]. As a limitation, we did not separate neutrophils and macrophages from BALF, due to challenges inherent in isolating alveolar neutrophils, but our analysis revealed that the 92% of the cells were neutrophils. Moreover, we cannot rule out that macrophages might also contribute to ET release in the lung. Additionally, it is presumable that cf-DNA in supernatants could be derived from different types of cell death other than ETosis. Thus, we analyzed co-localization of citrullinated histone H3 (Cit-H3)/DNA by immunofluorescence as a proxy to identify ET structures. Although the deimination of H3, catalyzed by peptidylarginine deiminase (PAD), is controversial concerning the trigger stimulus, strong evidence has shown that histones are citrullinated during NETosis induced by LPS [21,33,34]. It seems that histone deimination in neutrophils is induced in response to inflammatory stimuli instead of treatments that induce apoptosis [34], and that decondensed chromatin positive for histone citrullination occurs without caspase-3 cleavage, which is a prominent marker of cell death via apoptosis [35]. Our photomicrographs showed that LPS administration lead to an accumulation of ETs, which was mitigated by Gln treatment. Therefore, our in vitro findings suggest that the modulation of ET release by Gln in BALF cells stimulated with LPS could be a mechanism of action for protective effects of Gln in pulmonary ARDS. 

The classical mechanism of NET release, known as NETosis, is dependent on ROS generation. Nevertheless, the roles of ROS in dismantling the nuclear envelope and association of NET components remains unclear [24]. Some studies suggest that ROS directly promotes the morphologic changes observed during NET formation [36]. ROS may alternatively inactivate caspases, which inhibits apoptosis and favors autophagy. This leads to dissolution of cell membranes [37]. In this context, we also analyzed ROS formation in response to LPS stimulation in cells incubated in media with or without Gln. ROS production is routinely measured with DHR 123 using fluorescence plater reader assays, flow cytometry, or fluorescence microscopy. However, we chose to use fluorescence, based on previous studies on NET and ROS production [38,39]. Cells incubated in Gln-containing media exhibited less ROS formation after LPS stimulation than cells cultured without Gln. One of the mechanisms of action of Gln in ARDS involves enhancement of GSH content [10]. Thus, our data suggest that Gln may reduce ET formation by increasing GSH content and inhibiting ROS formation. Unfortunately, we were not able to demonstrate an influence of the presence of Gln on GR or GPx activity in vitro.

Since Gln is a very versatile amino acid, many different molecular pathways may be involved in its decreasing ET release in pulmonary ARDS. As previously discussed, the citrullination of histone H3 observed in our results seems to be related to specific proteins such as PAD4 [21,33,34,35]. Neutrophils isolated from mouse BALF underwent extensive H3 citrullination when exposed to LPS and high-mobility group box (HMGB)1 while treatment with HMGB1-neutralizing antibodies reduced NET release [40]. Additionally, evidence shows that the administration of Gln inhibits HMGB1 through increased expression of heat shock protein (HSP) 70 and, consequently, reduces lung damage in sepsis-induced lung injury [41]. Although this study evaluated the effect of Gln in an extrapulmonary ARDS model, we cannot rule out the possibility that Gln reduced HMGB1 in the present model of pulmonary ARDS, which contributes to a reduction in PAD4-mediated HMGB1 release in ETosis. Concerning HSP70, it is recognized that Gln is a potent expression enhancer of this chaperone [42]. A recent study found that upregulation of HSP70 protected against increased mitochondrial ROS release and pulmonary endothelial permeability induced by bacterial toxin exposure [43]. Additionally, administration of Gln in a model of sepsis-induced lung injury attenuated NF-kappaB transcription factor activation and proinflammatory cytokine expression [44]. The activation of NF-kappaB induced by LPS was also reduced by Gln in a mechanism involving the Akt/mTOR/IKK pathway [45]. Even though these studies differ from ours in terms of lung injury models and Gln administration route, it is possible that increased HSP70 may have contributed to reductions in ROS production, NF-kappaB activation, and proinflammatory cytokine release, all of which are known stimulis of ETosis.

In view of the foregoing, therapeutic strategies to reduce ETs formation in ARDS could be promising. However, the best agent to control ET release according to ARDS etiology and the pathway of ET formation as well as the role of macrophages and neutrophils in ARDS have yet to be established. Additionally, in experimental studies, the effects and mechanisms of Gln action have been studied in different ARDS models, mainly extrapulmonary [22,34,46]. However, the most common cause of ARDS is pneumonia (viral or bacterial) [1,2] and the results of Gln administration in pulmonary ARDS need to be better studied. The LPS dose administered in our model was based on a previous study, which showed ultrastructural and morphological changes, neutrophil infiltration in the lung parenchyma, and pulmonary mechanical compromise 24 h after induction of pulmonary injury. This is not a model of severe injury and does not lead to death [15]. 

The present study has some limitations. First, a model of pulmonary ARDS induced by a single intratracheal dose of *E. coli* LPS was used. Thus, our results cannot be extrapolated to other ARDS models or directly to the clinical setting. Second, mice received only a single dose of intravenous Gln 6 h after induction of lung damage. Thus, we do not know the effects that a different administration route or different timing of treatment might have in our model of pulmonary ARDS. Third, we analyzed in vivo data 24 hours after lesion induction, and, consequently, have no information about earlier or later effects. Fourth, since the gold standard marker of NETosis or the method of NET detection has not been established yet, we based our experiments on detecting ETs in previous studies evaluating NETosis [18,21,33,34,35,38,39]. Fifth, our first aim was to analyze the overall effects of Gln in alveolar-space cells without discriminating which cells would act to decrease ET formation. Our results revealed that the benefits of Gln use in ARDS might be related to ET modulation. This was the first step toward a detailed investigation on the behavior of the different alveolar-space cells, including macrophages and T-cells, during ETosis in a model of pulmonary ARDS. Sixth, in our study, Gln was analyzed but its metabolites were not analyzed. Thus, we cannot rule out possible effects of different metabolites and dosages in experimental ARDS. Seventh, a single dose of Gln was used. More research should be carried out to evaluate the effects of lower and higher doses of Gln in pulmonary ARDS. Eighth, lung damage was evaluated using light microscopy rather than electron microscopy. Lastly, it would be interesting to perform further studies evaluating Gln action in macrophages and neutrophils separately at different time points. 

## 5. Conclusions

In pulmonary ARDS, treatment with Gln reduces pulmonary functional and morphological impairment, inflammation, and ET release in the lung.
